# No benefit of antidepressants in inpatient treatment of depression. A longitudinal, quasi-experimental field study

**DOI:** 10.1007/s00213-023-06417-4

**Published:** 2023-08-01

**Authors:** Reinhard Maß, Kerstin Backhaus, Katharina Lohrer, Michael Szelies, Bodo K. Unkelbach

**Affiliations:** Center for Mental Health Marienheide, Leppestr. 65-67, 51709 Marienheide, Germany

**Keywords:** Depression, Inpatient treatment, Antidepressants, Psychotherapy

## Abstract

**Rationale:**

Antidepressants (AD) are mostly considered indispensable for the treatment of major depression. The vast majority of depressive inpatients are treated with AD. However, there is a growing body of studies indicating that the effectiveness of AD is greatly overestimated due to methodological issues with the AD efficacy studies (e.g., publication bias, unintentional unblinding, confusion between withdrawal symptoms and relapse).

**Objectives:**

The benefit of the additional use of AD in the inpatient treatment of depression with intensive cognitive-behavioral therapy (CBT) has been investigated in a naturalistic design.

**Methods:**

Depressiveness was assessed using the Beck Depression Inventory (BDI-II) during a preliminary interview (T0), at admission (T1), at discharge (T2), and at a 6-month follow-up (T3). Two study phases were compared: During Phase A, AD were recommended in accordance with the German guideline. In Phase B, AD were no longer recommended, and they were only prescribed upon explicit request from patients. In phase A (*N* = 574), 60.3% of all patients were taking AD at discharge. In Phase B (*N* = 424), 27.9% of patients were on AD at discharge. Apart from the difference in AD usage, the two treatment conditions were similar, and the samples did not significantly differ in terms of age, sex, diagnoses, history of suicide attempts, comorbid anxiety disorders, and unemployment.

**Results:**

In both study phases, BDI-II scores were strongly decreased at T2 and T3, respectively, compared with T1. The BDI-II scores of the two phases did not differ at any of the measurement time points. Depression changes were similar in both phases. In sequential multiple regression analyses with the total sample, AD were no significant predictors for the reduction of depression at either T2 or T3.

**Conclusions:**

The inpatient CBT was effective in depression. The effectiveness of CBT is not improved by the additional use of AD. The current prescribing practices of AD should be questioned.

## Introduction

Numerous studies and meta-analyses support the short-term effectiveness of antidepressants (AD) for treating severe depression (e.g., Kirsch et al. 2008; Tondo et al. 2013; Cipriani et al. 2018; Stone et al. 2022). The prescription AD is increasing year over year (Kendrick 2021), although the prevalence of depression appears to be constant (Patten et al. 2016). In German psychiatric inpatient treatment, AD use is the norm; in a multicenter study including more than 3000 depressed inpatients (Härter et al. 2004), 93.5% took AD. The use of AD must be viewed critically not only because of the harmful side effects (e.g., increased suicidality; Hengartner and Plöderl 2019) but also because discontinuation reactions and rebound effects often cause long-term use of AD (Davies et al. 2019), whose therapeutic benefit is often overestimated for several reasons (Holper and Hengartner 2020; Turner et al. 2022; Stone et al. 2022). According to some naturalistic observational studies, AD may worsen the prognosis in depression (Hengartner et al. 2018, 2019). The value of AD in preventing relapse is based on studies that likely mix relapse and withdrawal symptoms, and therefore have limited value (Hengartner 2020).

A naturalistic multicenter study (Zeeck et al. 2015) included 301 psychosomatic inpatients with depression; 52.5% were taking AD at discharge. Post-hoc analyses (Zeeck et al. 2016) showed that AD were not a predictor of the course of depression in the follow-up period.

In another study on the effects of psychotherapy (Dinger et al. 2015), 44 depressed inpatients were treated for eight weeks and followed up one month and six months later. At discharge, 51.4% of the patients were taking AD. The depressive symptoms significantly decreased from admission to discharge and slightly increased between discharge and 6-month follow-up. Again, AD did not affect treatment outcomes.

Recently, a large meta-analysis on the efficacy of cognitive-behavioral therapy (CBT) conducted by Cuijpers et al. (2023) analyzed over 400 trials with more than 52,000 patients. The study found no evidence that combined treatment of CBT and AD is superior to CBT monotherapy.

In our own study (Maß et al. 2019) involving 574 psychiatric inpatients suffering from depression, recruited between October 2012 and December 2017, depression greatly decreased from admission to discharge as well as to 6-month follow-up. All patients received psychotherapeutic treatment. Additional treatment with AD was received by 364 patients (60.3%) at discharge. Post-hoc analyses showed that the additional use of AD had no effect at either discharge or follow-up. Based on these observations, since January 2018, AD were no longer a part of our treatment concept. In all other respects, the treatment concept remained the same, and data collection also continued unchanged. This enables the division of the observation period into two sections: “Phase A” (October 2012 to December 2017, see Maß et al. 2019), in which AD were still part of the treatment concept, and “Phase B” (January 2018 to June 2021), newly presented here, in which they were not.

The present study aims to compare phases A and B. If the modest short-term effectiveness demonstrated in several meta-analyses (e.g., Cipriani et al. 2018) translates into real-world effectiveness during routine practice, we anticipate superior treatment outcomes in phase A relative to phase B, as well as for patients who use AD compared to non-users.

## Methods

### Sample

All patients were recruited on the “Aaron T. Beck” ward, a general psychiatric ward with 20 treatment places at the Center for Mental Health Marienheide. The Center has a rather rural catchment area. The therapeutic offer is aimed at adults with all forms of mental disorders except organic brain disorders and addiction. Almost all patients had moderate or severe unipolar depression, and some had comorbid disorders. Preliminary interviews were conducted with some of the patients, from which the waiting list control groups were formed. All patients admitted to the ward between October 2012 and June 2021 were included in the study. In phase A and phase B, 574 and 424 cases were recruited, respectively. There were no exclusion criteria. Hospitalisation was due to the severity of clinical conditions (high risk of suicide, inability to cope with everyday life, inability to work, and unsuccessful outpatient treatment).

Age and sex distribution were the similar in both phases. Phase A: $\overline{x }$ = 37.3 years (*SD* = 13.1), 250 men (43.6%), 324 women (56.4%); phase B: $\overline{x }$ = 37.9 years (*SD* = 13.6), 183 men (43.2%), 241 women (56.8%); *t* = 0.793, *df* = 996, *p* = 0.793; χ^2^ = 0.015, *df* = 1, *p* = 0.901. There was a significant difference in the mean treatment duration (only regularly discharged patients); phase A: $\overline{x }$ = 59.7 days (*SD* = 21.0); phase B: $\overline{x }$ = 55.2 days (*SD* = 18.7); *t* = 3.278, *df* = 865, *p* < 0.001. The waiting time between preliminary interview and admission was similar; phase A: $\overline{x }$ = 39.5 days (*SD* = 34.4); phase B: $\overline{x }$ = 35.4 days (*SD* = 28.3); *t* = 1.300, *df* = 391, *p* = 0.194.

All diagnoses were assigned according to the criteria of the ICD-10. All patients received a diagnosis of major depression. Many patients received additional diagnoses, such as personality disorders, anxiety disorders, obsessive–compulsive disorders, eating disorders, psychoses, and PTSD. For the purposes of the analyses, the patients were divided into three groups according to their diagnoses. The DEP group consisted of all cases with a diagnosis of major depressive disorder. The PER group comprised all cases with a personality disorder (mainly borderline disorder) and comorbid major depression. The OTH group included all other disorders (see above), again with comorbid major depression. Secondary diagnoses of anxiety disorders (e.g., panic disorder) were allowed in all three groups. The relative size of the three diagnostic groups was similar in both phases (χ^2^ = 0.052, *df* = 2, *p* = 0.974); phase A: DEP 370 (64.5%), PER 112 (19.5%), OTH 92 (16.0%); phase B: DEP 276 (65.1%), PER 82 (19.3%), OTH 66 (15.6%). If multiple admissions occurred within an episode of illness, treatments were combined and considered a single case. If a recurrence of disease after remission led to readmission, it was considered a new case. In phase A, this was true for 38 of the 574 cases (6.6%), and the median interval between treatments was 464 days. In phase B, 20 of the 424 cases (4.7%) were readmissions (median of the interval: 373 days). The two phases did not differ in the proportion of multiple admissions (χ^2^ = 1.614, *df* = 1, *p* = 0.204). The proportion of DEP patients with recurrent depression was 57.3% in phase A and 60.5% in phase B (χ^2^ = 0.672, *df* = 1, *p* = 0.412).

The samples may differ in additional characteristics that are relevant for the outcome. Have et al. (2017) and Buckman et al. (2021) have shown that depressive patients with comorbid anxiety disorders are more likely to receive antidepressants and have a worse prognosis. Suicidality (Reutfors et al. 2021) and unemployment (Buckman et al. 2022) are also prognostically relevant factors. However, no significant differences were found in the frequencies of comorbid anxiety disorders (phase A: 19.5%, phase B: 17.7%, χ^2^ = 0.533, *df* = 1, *p* = 0.466), history of suicide attempts (phase A: 16.0%, phase B: 12.5%, χ^2^ = 2.444, *df* = 1, *p* = 0.118), and unemployment (phase A: 30.8%, phase B: 26.4%, χ^2^ = 2.317, *df* = 1, *p* = 0.128).

### Treatment concept

The treatment concept of the “Aaron T. Beck” ward has already been described in detail (Maß et al. 2019, 2022). The ward is oriented towards CBT, with a systemic-biographical understanding of the etiology and maintenance of mental disorders (Zarbock 2017). Upon admission, an individualized model of predisposing, precipitating, and maintaining factors was developed, which formed the basis for treatment goals and strategy. Depression is viewed as a response to acute and chronic psychosocial stresses (e.g., workload, partnership problems, or interpersonal conflicts) combined with specific vulnerabilities (e.g., dysfunctional cognitive beliefs, deficits of social or emotional competence, or somatic problems) that lead to decompensation. Treatment is aimed at developing better coping mechanisms. The main components of the treatment concept are two individual psychotherapeutic sessions per week, plus group therapies (e.g., depression group, social skills training, mindfulness, and embodiment) and adjuvant therapies (e.g. PMR, occupational therapy, and physiotherapy). AD were administered in accordance with the manufacturers' recommendations. Phase A: Upon admission, 118 (20.6%) of the 574 patients received an AD at a standard dosage, 82 (14.3%) at an increased dosage, 44 (7.7%) at a reduced dosage, and 61 (10.7%) received more than one AD. Upon discharge, 136 (23.7%) received an AD at a standard dosage, 110 (19.2%) at an increased dosage, 37 (6.4%) at a reduced dosage, and 59 (10.3%) received more than one AD. Phase B: Upon admission, 59 (13.9%) of the 424 patients received an AD at a standard dosage, 62 (14.6%) at an increased dosage, 40 (9.4%) at a reduced dosage, and 31 (7.3%) received more than one AD. Upon discharge, 39 (9.2%) received an AD at a standard dosage, 27 (6.4%) at an increased dosage, 42 (9.9%) at a reduced dosage, and 11 (2.6%) received more than one AD.

### The investigation phases A and B

In phase A, the treatment concept included the recommendation of AD for severe cases of depression, in accordance with the current guideline. In phase B, however, contrary to the guideline, no AD were recommended and were only prescribed at the explicit request of the patient. Most patients chose not to take AD (72.6% at discharge). Apart from this difference, the treatment concept remained identical in both phases as described above.

### Instruments

All depression diagnoses were validated using the International Diagnostic Checklists (IDCL; Hiller et al. 1995). When necessary, other diagnoses were also checked with the IDCL.

Depressiveness was assessed with the revised Beck Depression Inventory (BDI-II; Beck et al. 1996). This self-report instrument is an international standard in depression research.

### Procedure

The BDI-II was administered to all patients at up to four time points: at the preliminary interview (T0) which, however, was only conducted with some of the patients; at admission (T1); at discharge (T2); and at the follow-up six months after discharge (T3). At discharge, all patients were asked to participate follow-up. All regularly discharged patients agreed. They received a letter after six months with the BDI-II, some additional questions for the retrospective evaluation of the treatment, and a stamped, addressed envelope.

### Statistics

Statistical analyses were performed using the Statistical Package for the Social Sciences (SPSS, version 22). Independent-samples t-tests, paired-samples t-tests, a two-way ANOVA, Pearson correlations, χ^2^ tests, effect sizes for repeated measures (Morris and DeShon 2002), and sequential multiple regression analyses (SMRA) were calculated. Hypothesis testing was two-tailed.

### Ethics

The study is provided with the number 2014346, with a positive vote by the Ethics Committee of the Medical Association Nordrhein/Germany.

## Results

Table 1 shows, per phase and diagnostic group, the AD prescribed at admission and discharge, summarized by substance group.

**Table 1:** Antidepressants in both study phases

		DEP		PER		OTH	
		Admission	Discharge	Admission	Discharge	Admission	Discharge
Phase A	SSRI	108 (29.2%)	95 (25.7%)	20 (17.8%)	19 (17.0%)	25 (27.2%)	22 (23.4%)
	TCA	67 (18.1%)	60 (16.2%)	13 (11.6%)	15 (13.4%)	11 (12.0%)	7 (7.6%)
	SNRI	49 (13.2%)	82 (22.2%)	13 (11.6%)	17 (15.2%)	11 (12.0%)	17 (18.5%)
	Agomelatine	25 (6.8%)	46 (12.4%)	7 (6.3%)	13 (11.6%)	4 (4.3%)	3 (3.3%)
	other AD	4 (1.0%)	5 (1.3%)	2 (1.8%)	1 (0.9%)	–	–
	no AD	161 (43.5%)	126 (34.1%	63 (56.3%)	55 (49.1%)	47 (51.1%)	47 (51.1%)
Phase B	SSRI	53 (19.2%)	24 (8.7%)	23 (28.0%)	10 (12.2%)	14 (23.3%)	9 (15.0%)
	TCA	51 (18.5%)	35 (12.7%)	13 (15.9%)	9 (11.0%)	7 (11.7%)	5 (8.3%)
	SNRI	34 (12.0%)	15 (5.4%)	10 (12.2%)	8 (9.8%)	7 (11.7%)	6 (10.0%)
	Agomelatine	7 (2.5%)	7 (2.5%)	3 (3.7%)	2 (2.4%)	–	–
	other AD	2 (0.7%)	–	–	–	1 (1.7%)	1 (1.7%)
	no AD	149 (54.0%)	204 (74.0%)	43 (52.4%)	58 (70.7%)	38 (63.3%)	46 (76.7%)

Percentages sum to > 100 because some patients were taking more than one AD

*SSRI* selective serotonin reuptake inhibitors, *TCA* tri- and tetracyclic AD, *SNRI* serotonin and norepinephrine reuptake inhibitors, *DEP* Depression, *PER* depression with comorbid personality disorder, *OTH* depression with comorbid other mental disorder

To determine AD effects, patients were divided into five AD groups (see Maß et al. 2019):

- group “*no AD*” did not take AD at any time during treatment;
- group “*AD prescribed*” was not taking AD at admission, received AD during the treatment, and was thus discharged;
- group “*AD discontinued*” was taking AD at admission, which were discontinued during treatment;
- group “*AD maintained*” was taking AD at admission, which were maintained without change;
- group “*AD changed*” was taking AD at admission for which changes were made during treatment (agent, dose, or augmentation).

As expected, samples differed significantly with respect to AD groups (χ^2^ = 127.337, *df* = 4, *p* < 0.001). Phase A: *no AD* 190 (33.1%), *AD prescribed* 78 (13.6%), *AD discontinued* 37 (6.4%), *AD maintained* 124 (21.6%), *AD changed* 145 (25.3%); phase B: *no AD* 224 (52.8%), *AD prescribed* 8 (21.9%), *AD discontinued* 84 (19.8%), *AD maintained* 53 (12.5%), *AD changed* 55 (13.0%). Thus, 227 (39.5%) patients in phase A and 308 (72.6%) in phase B were discharged from treatment without AD (χ^2^ = 107.395, *df* = 1, *p* < 0.001).

The BDI-II scores of the patients did not differ between the two phases at any time (see Table 2). A two-way ANOVA with phase (A/B) and AD at T1 (y/n) as factors, and BDI-II score at T1 as the dependent variable yielded a significant effect of AD at T1 (patients taking AD had scores approximately three points higher than patients not taking AD; F [1, 994] = 21.634, *p* < 0.001); the effect of phase (F [1, 994] = 1.775, *p* = 0.183) and the interaction effect phase × AD (F [1, 994] = 0.012, *p* = 0.912) were not significant.

**Table 2:** BDI-II scores at the four measurement time points, comparison of phases A and B

BDI-II total score	Phase A $\overline{x }$ (SD), N	Phase B x̄ (SD), N	*t*	*df*	*p*
T0 Preliminary Interview	34.7 (9.5), 181	35.4 (9.1), 201	−0.740	380	.460
T1 Admission	31.4 (11.1), 574	32.1 (10.3), 401	−1.024	973	.306
T2 Regular Discharge	13.2 (10.2), 489	13.3 (10.7), 378	−0.090	865	.928
T3 Follow-up	18.3 (12.7), 306	18.6 (12.4), 278	−0.328	582	.743

Table 3 shows the changes in depressiveness between the measurement time points in phase A and phase B (all changes are statistically significant, not shown here). Unpaired samples t-tests revealed similar mean BDI-II difference scores in both phases. The effect sizes d_RM_ are comparable in both phases: weak effects (−0.4) for the decreases in BDI-II scores during the waiting period, strong effects (−1.6 and −1.7, respectively) for the decreases from admission to discharge, medium effects (0.6) for the increase in the half-year between discharge and follow-up, and still strong effects for the decrease from admission to follow-up (−1.1 and −1.3, respectively). The length of waiting time between preliminary interview and admission was not related to the change in BDI-II (phase A: *r* = 0.03, *p* = 0.676; phase B: *r* = −0.04, *p* = 0.596).

**Table 3:** Changes in BDI-II scores over time with effect sizes; comparison of phases A and B

		BDI-II differences $\overline{x }$ (SD)	d_RM_	*t*	*df*	*p*
Change during waiting time (T1 minus T0)	Phase A (*N* = 181)	−2.50 (7.10)	−0.375	−0.101	391	.919
	Phase B (*N* = 212)	−2.43 (6.23)	−0.410			
Outcome at discharge (T2 minus T1)^a^	Phase A (*N* = 489)	−18.26 (10.85)	−1.629	0.283	865	.777
	Phase B (*N* = 378)	−18.48 (11.08)	−1.707			
Change during follow-up time (T3 minus T2)^a^	Phase A (*N* = 306)	5.39 (11.33)	0.555	−0.203	589	.839
	Phase B (*N* = 285)	5.58 (11.01)	0.557			
Outcome at follow-up (T3 minus T1)^a^	Phase A (*N* = 306)	−12.87 (12.97)	−1.060	0.302	589	.604
	Phase B (*N* = 285)	−13.40 (11.73)	−1.254			

^a^ only regularly discharged patients

In addition, (regularly discharged) patients were divided into three groups at T2 and T3: “non-responders” (decrease in BDI-II score from T1 to T2 or T3 by less than 50%), “responders” (decrease by at least 50%), and “remitted patients” (BDI-II score of T2 or T3 12 points or less). At discharge (T2), there were 28.4% non-responders, 16.0% responders, and 55.6% remitted patients in phase A, and 28.3% non-responders, 14.8% responders, and 56.9% remitted patients in phase B (χ^2^ = 0.239, *df* = 2, *p* = 0.887). At follow-up (T3), there were 49.7% non-responders, 8.8% responders, and 41.5% remitted patients in phase A, and 48.8% non-responders, 12.3% responders, and 38.9% remitted patients in phase B (χ^2^ = 1.945, *df* = 2, *p* = 0.378).

Of the regularly discharged patients who received AD at T2, 52.9% were non-responders, 12.0% were responders, and 35.0% were remitted at T3; among patients who did not receive AD at T2, 46.1% were non-responders, 9.1% were responders, and 44.8% were remitted (χ^2^ = 6.056, df = 2, *p* = 0.048).

A SMRA was calculated over the combined sample (regularly discharged patients) with the dependent variable “BDI-II difference T2 minus T1” and the following predictors: BDI-II at T1, age (years), gender (m/f), duration of treatment (days), unemployment (y/n), suicide attempts (y/n), co-morbid anxiety disorder (y/n), study phase (A/B), AD at admission (y/n), AD at discharge (y/n). The decrease of BDI-II from T1 to T2 was predicted by BDI-II at T1 (the higher the score, the larger the decrease; F [1, 865] = 344.456, *p* < 0.001), unemployment (related to lower decrease; F [1, 861] = 3.927, *p* = 0.048), and suicide attempts (related to lower decrease; F [1, 860] = 3.024, *p* = 0.082). The other predictors did not show any significant effect.

A further SMRA was calculated over the combined sample with the dependent variable “BDI-II difference T3 minus T1” and the following predictors: BDI-II at T1, BDI-II at T2, age, gender, duration of treatment, unemployment, suicide attempts, co-morbid anxiety disorder, study phase, AD at admission, AD at discharge. The decrease of BDI-II from T1 to T3 was predicted by BDI-II at T1 (the higher the score, the larger the decrease; F [1, 589] = 137.136, *p* < 0.001), BDI-II at T2 (the higher the score, the lower the decrease; F [1, 588] = 117.121, *p* < 0.001), age (the older the patient, the lower the decrease, F [1, 587] = 127.706, *p* < 0.001), duration of treatment (the longer the treatment, the larger the decrease; F [1, 585] = 3.384, *p* = 0.066) and unemployment (related to lower decrease; F [1, 584] = 1.174, *p* = 0.099) The other predictors did not show any significant effect.

In phase B, 268 of the patients were asked at T1 as to the extent of help they expected from psychotherapy or medication for their current problems. Agreement with two independent statements was recorded using a four-point scale: “I believe that medication will help me overcome my current problems.” and “I believe that psychotherapy will help me overcome my current problems.” (I agree totally/ mostly/ somewhat/ not at all). Of all patients surveyed, 96.6% (97.9% of group DEP) indicated that they expected help totally or mostly from psychotherapy, whereas only 34.7% of all patients (31.7% of group DEP) hoped totally or mostly for help from medication.

In phase A, the proportion of dropouts at T2 was 14.8% (including four suicides by patients on AD), whereas phase B showed 10.8% dropouts and no suicide (χ^2^ = 3.352, *df* = 1, *p* = 0.067. In most cases, the cause of the drop-out was treatment discontinuation due to a loss of motivation to continue with psychotherapy. In some cases, treatments were terminated by the staff due to serious rule violations (e.g., drug abuse). The drop-out from T2 to T3 was 37.4% in phase A and 24.6% in phase B (χ^2^ = 16.148, *df* = 1, *p* < 0.001). The reasons for the drop-out at T3 are not known.

At follow-up, an open-ended question was asked about the retrospectively most important components of treatment. The answers were assigned to categories, including the two categories “psychotherapy” and “pharmacotherapy”. Of the patients in phase A, 81.0% reported that psychotherapy was among the most important components of treatment for them, compared with 78.2% in phase B (χ^2^ = 0.715, *df* = 1, *p* = 0.398). In contrast, 2.3% of participants in phase A reported that pharmacotherapy was important to them; in phase B, this proportion was 2.8% (χ^2^ = 0.161, *df* = 1, *p* = 0.688). Furthermore, patients were asked to answer the sentence “I am satisfied with the outcome of the treatment on ward ‘Aaron T. Beck’ from today’s point of view” on a scale with the four levels “true exactly” (3 points), “true mostly” (2 points), “true somewhat” (1 point), and “not true at all” (0 points). Mean satisfaction with treatment outcome at T3 was 2.38 (*SD* = 0.77) points in phase A and 2.37 (*SD* = 0.72) points in phase B; *t* = 0.257, *df* = 584, *p* = 0.797. There was no significant difference in treatment satisfaction between the patients discharged with AD and those discharged without AD in neither of the study phases or in the combined sample.

Clinical experience with the discontinuation of AD. In our study, a total of 121 patients discontinued AD during treatment. Unfortunately, we do not have detailed documentation on the withdrawal symptoms. Discontinuation was carried out in one step or over several weeks, depending on circumstances such as, e.g., the type of antidepressant, dosage, duration of intake, and was usually completed well before discharge. Many patients described the usual transient withdrawal symptoms (e.g., nausea, imbalance, headache, insomnia) but were mostly able to tolerate them; if necessary, the next dose reduction was postponed. The hospital setting with frequent physician contacts was helpful because it provided patients with a sense of security.

## Discussion

The omission of AD as part of the inpatient treatment concept demonstrated no disadvantages. There is no evidence of an additional benefit of AD. Thus, the central hypothesis of this study is to be rejected. BDI-II scores declined sharply in both phases until discharge and, despite a slight increase, were still significantly lower at follow-up than at admission. Furthermore, the previously (Maß et al. 2019) described treatment effects are widely replicated.

Some superiority of treatment is evident in phase B: (1) The treatments in phase B were 4½ days shorter; (2) in phase A, the proportion of drop out was greater than in phase B; (2) phase B shows somewhat higher effect sizes for the decreases in depression at T2 and T3.

Typically, patients who are willing to engage in intensive psychotherapy are admitted to the ward “Aaron T. Beck”. This was the case for the majority of all eligible patients. The preference for psychotherapy is not a peculiarity of our sample; rather, psychotherapy for depression is also preferred in the general population (Angermeyer et al. 2017). Hence, the approach without AD in phase B seems to be more in line with patients’ wishes, which made it easier for them to engage in treatment and to participate in T2 and T3.

The results presented here appear to contradict previous studies, suggesting that AD are effective, e.g., the above-mentioned meta-analysis by Cipriani et al. (2018), which is based on 522 randomized controlled trials (RCTs) attracted attention. All the 21 AD considered in this study were more efficacious than placebo. However, this meta-analysis has been criticised for several methodological flaws (Hengartner and Plöderl 2018; McCormack and Korownyk 2018). Recently, an individual participant data analysis of 232 RCTs reported by Stone et al. (2022) showed that only 15% of patients seem to have a pharmacological benefit from AD. In general, significant methodological problems have been highlighted in AD efficacy trials (Hengartner 2017; Kirsch 2019; Munkholm et al. 2019). A major problem is the breaking of the double-blind condition in RCTs (Margraf et al. 1991; Moncrieff et al. 2004; Baethge et al. 2013) which seriously questions the validity of these studies. Stone et al. (2022, p. 8) pointed out that the 15% proportion of patients who seem to have a pharmacological benefit could also be explained by the effects of functional unblinding.

The question arises as to why we did not observe a placebo effect of the AD in our study. There are two complementary hypotheses to explain this: (1) A large proportion of the patients in our sample had the experience of becoming or remaining depressed despite taking AD; (2) The psychosocial model of depression described above, developed at the beginning of treatment, helps patients understand their illness in the context of their current life situation and personal history. The strength of the placebo effect is heavily influenced by situational conditions, with patient expectations being a significant component (Enck et al. 2013). As the aforementioned factors leave little room for expecting help from AD, this may have prevented a placebo effect. Furthermore, our sample consists of inpatients who primarily seek psychotherapeutic assistance, which may result in differing expectations compared to outpatients seeking psychopharmacological treatment and participating in RCTs.

Our findings are in line with Blom et al. (2007). They compared four groups of depressed patients treated with either an AD (nefazodone), interpersonal psychotherapy (IPT), a combination of IPT and AD, or a combination of IPT and a placebo. No difference was noted in outcome between IPT with vs. without AD; at the same time, the combination of IPT and AD was associated with a stronger treatment effect than with AD alone. It was concluded that a combination of psychotherapy and pharmacotherapy is superior to monotherapy only when pharmacotherapy is supplemented with psychotherapy but not vice versa. These results have recently been supported by Cuijpers et al. (2023), confirming that the combination of AD and CBT is more effective than AD alone in both the short and long term, but not superior to CBT alone. Therefore, adding AD to CBT does not confer any treatment benefits, but only increases the burden of side effects.

Unfortunately, there are only very few RCTs with inpatients. The outcomes of inpatient treatments are difficult to compare with RCTs and meta-analyses involving outpatients (e.g., Cipriani et al. 2018; Stone et al. 2022) due to the complex and strong effects of the treatment programs received by inpatients. The lack of difference between inpatient treatments with and without AD can be explained by a ceiling effect. However, all the patients investigated by Blom et al. (2007) and almost all of the patients in Cuijpers et al.’s (2023) meta-analysis were outpatients, which makes a ceiling effect in these studies unlikely. Nonetheless, the results shown there are similar to those of our study. This suggests that a ceiling effect does not play a significant role in our results.

We did not observe any recurrences or rebounds related to AD discontinuation (except one patient manifesting a hypomanic syndrome for the first time during discontinuation, which remitted before discharge without intervention). Though this observation seems to contradict previous works (e.g., Kato et al. 2021), there is a recent discussion about a possible confusion between discontinuation symptoms vs. depressive symptoms (Récalt and Cohen 2019; Hengartner 2020).

### Criticism of the German guideline

The current German guideline for depression (Bundesärztekammer et al. 2022) is strongly oriented towards the use of AD. According to the guideline, AD and psychotherapy are considered equivalent forms of treatment for moderate depression, and Patients with severe, chronic, or recurrent depression should be treated with a combination of AD and psychotherapy. This is because combination therapy is considered more effective than either pharmacotherapy or psychotherapy alone. Our findings show that a deviation from this recommendation does not lead to worse outcomes. Something similar has been shown in the above-mentioned multicentre study (Zeeck et al. 2015, 2016); up to 12 months after discharge, guideline-compliant post inpatient treatment resulted in the same outcomes as a treatment that did not comply with the guideline (Weiß et al. 2020). The authors conclude that guideline recommendations should not be considered rigid rules. However, even if the recommendations are not legally binding, they have considerable influence. Mendel et al. (2010) showed that psychiatrists prescribe AD to their patients much more often in case of depression than they would take it themselves. One reason for this is the concern of being vulnerable to legal action if complications arise (e.g., suicide attempt) if the treatment deviates from the guideline. It is therefore all the more important that guideline recommendations take into account the current state of research. Psychotherapy, particularly CBT, seems to be a sufficient treatment for depression and is not improved by the use of AD (Blom et al. 2007; Maß et al. 2019; Cuijpers et al. 2023). The treatment guidelines should therefore be updated accordingly. However, this could be hindered by the fact that many authors of the guideline have financial relationships with the pharmaceutical industry. This fact leads to a mixture of scientific and commercial perspectives and has been repeatedly subjected to severe criticism (e.g., Fava 2016; Hengartner 2017; Shorter 2021).

### Limitations

Pharmacotherapy at follow-up (T3) is largely unknown. Only a subsample of the DEP group in phase B (*N* = 36) was asked about AD at T3. Of the patients, 28 had been discharged without AD and were not taking AD at T3. Five had been admitted without AD but were taking AD at T3. The three remaining patients were taking AD both at discharge and at T3. A total of eight patients taking AD at T3 had a mean BDI-II score of 24.3 points (*SD* = 13.5), and the 28 patients without AD had a mean score 15.6 points (*SD* = 14.1). This indicates that most patients discharged without AD continued not to take AD in the six months after discharge; those taking AD at T3 did not seem to benefit. Given the small number of cases, it is not certain how generalizable these data are.

This work is not formally a field experiment, because the change of treatment concept in phase B was a therapeutic decision, not part of an experimental design. In fact, however, the two treatment concepts correspond to two phases of an experiment in which the use of AD is the independent variable and BDI-II is the dependent variable.

In addition to the advantages of high ecological and external validity, naturalistic studies have the disadvantage of reduced internal validity. Sample sizes differ, and comparison groups were not formed by random assignment. Recruitment, treatment duration, pharmacotherapy, etc., did not follow an experimental design. The primary outcome measure (BDI-II) was a self-rating instrument. Its validity depends on the ability to introspect; in some (rare) cases, introspection was impaired (possibly as a result of depression) so that the BDI-II score underestimated the true extent of depressiveness. Unfortunately, further information on depression (duration of the index episode, age at onset of illness, number of previous episodes) was not systematically recorded in this study.

The present results confirm our previous findings that AD do not benefit the inpatient treatment of depression. However, the data were collected in a single ward where intensive psychotherapy is administered. While there is currently no evidence that our patients differ from those of other clinics in rural areas, it would be beneficial to replicate this investigation in other clinics to establish the generalizability of our findings.
